# Orbital tumours

**DOI:** 10.1186/1470-7330-15-S1-O27

**Published:** 2015-10-02

**Authors:** Vincent Chong

**Affiliations:** 1 Department of Diagnostic Radiology, National University Hospital, National University Health System, Singapore 119074

## 

The approach to the accurate diagnosis of orbital lesions requires initial identification of the space of origin. For descriptive purposes, the orbit has been divided into the following parts: globe, intraconal and extraconal spaces [1]. As each space has unique contents, the diagnostic possibilities can to some extent be predicted accordingly. Such an approach works fairly well in clinical practice. However, some common orbital lesions such as inflammatory pseudotumour, lymphoma and metastatic disease typically affect multiple spaces. Under such circumstances, the pattern of involvement together with clinical information can often provide a reasonable tentative diagnosis.

An accurate histological prediction of a lesion is often difficult as many lesions share common imaging features. On the other hand, there are lesions with typical imaging features which render histological confirmation unnecessary. At times the delineation of disease extent is the most important role of imaging such as in the case of staging of head and neck malignancies. Radiologists should therefore be familiar with pertinent anatomical knowledge required for both tumour staging and surgical planning.

## Extraconal lesions

Epithelial tumours represent 50% of the masses involving the lacrimal gland. The remaining lesions are due to lympho-inflammatory lesions. Pleomorphic adenomas are the most common benign epithelial tumours. Adenoid cystic and mucoepidermoid carcinomas are the most common malignant neoplasms. Dermoid cysts are not true lacrimal tumours but arise from rest cells located in the orbit.

## Intraconal lesions

Optic nerve meningiomas are usually seen in middle age women [2]. On contrast enhanced CT or MRI meningiomas appear as tubular thickening or localised eccentric expansions. These tumours retain the same signal intensity as brain tissue on most pulse sequences and shows intense enhancement. The tramline sign may also be seen.

Optic nerve gliomas are benign tumours usually seen in childhood. CT or MRI shows fusiform thickening of the optic nerve. Tumours may show variable enhancement. On T1-weighted images, the tumour is isointense with white matter but the T2 signals are more variable.

Orbital schwannomas may arise from the III, IV, V1 or VI cranial nerves. They are more commonly seen in the intraconal space but may be seen anywhere in the orbit. On CT they appear sharply demarcated, oval or fusiform.

## Multiple compartment lesions

Of all patients with orbital lymphoma, up to 75% have systemic disease [4]. Lymphomas are homogeneous masses of relatively high density with sharp margins. Generally these lesions mould themselves without eroding or enlarging the orbit.

Plasmacytomas are closely related to lymphomas. Myelomas may affect the orbit and display the same spectrum of findings as in lymphomas. Masses maybe lobulated, well defined with or without bone destruction. They may also display intense enhancement.

Metastatic disease in the orbits can be seen in the eye (choroidal metastasis), optic nerve, intraconal, conal and extraconal spaces [5].

Pseudotumours usually affect more than one orbital space. For descriptive purposes, pseudotumours may be classified into the following types: 1) diffuse, 2) lacrimal & dacrocystitis, 3) myositis, 4) periscleritis, 5) perineuritis, and 6) Toloso-Hunt Syndrome
